# Assessing the capacity for newborn resuscitation and factors associated with providers’ knowledge and skills: a cross-sectional study in Afghanistan

**DOI:** 10.1186/1471-2431-13-140

**Published:** 2013-09-10

**Authors:** Young Mi Kim, Nasratullah Ansari, Adrienne Kols, Hannah Tappis, Sheena Currie, Partamin Zainullah, Patricia Bailey, Richard Semba, Kai Sun, Jos van Roosmalen, Jelle Stekelenburg

**Affiliations:** 1 Jhpiego/USA, an affiliate of Johns Hopkins University, 1615 Thames Street, Baltimore, MD 21231, USA; 2 Jhpiego/Afghanistan, an affiliate of Johns Hopkins University, Kabul, Afghanistan; 3 Johns Hopkins School of Public Health (JHSPH), Baltimore, MD, USA; 4 FHI 360 and Averting Maternal Death & Disability, Durham, NC, USA; 5 Johns Hopkins Medical Institute (JHMI), Baltimore, MD, USA; 6 Free University, Amsterdam, The Netherlands; 7 Department of Obstetrics & Gynecology, Leeuwarden Medical Center, Leeuwarden, The Netherlands

**Keywords:** Intrapartum hypoxia, Birth asphyxia, Emergency obstetric care, Neonatal mortality, Newborn resuscitation, Afghanistan, Lower income countries

## Abstract

**Background:**

Resuscitation with bag and mask is a high-impact intervention that can reduce neonatal deaths in resource-poor countries. This study assessed the capacity to perform newborn resuscitation at facilities offering comprehensive emergency obstetric and newborn care (EmONC) in Afghanistan, as well as individual and facility characteristics associated with providers’ knowledge and clinical skills.

**Methods:**

Assessors interviewed 82 doctors and 142 midwives at 78 facilities on their knowledge of newborn resuscitation and observed them perform the procedure on an anatomical model. Supplies, equipment, and infrastructure were assessed at each facility. Descriptive statistics and simple and multivariate regression analyses were performed using STATA 11.2 and SAS 9.1.3.

**Results:**

Over 90% of facilities had essential equipment for newborn resuscitation, including a mucus extractor, bag, and mask. More than 80% of providers had been trained on newborn resuscitation, but midwives were more likely than doctors to receive such training as part of pre-service education (59% and 35%, respectively, p < 0.001). No significant differences were found between doctors and midwives on knowledge, clinical skills, or confidence in performing newborn resuscitation. Doctors and midwives scored 71% and 66%, respectively, on knowledge questions and 66% and 71% on the skills assessment; 75% of doctors and 83% of midwives felt very confident in their ability to perform newborn resuscitation. Training was associated with greater knowledge (p < 0.001) and clinical skills (p < 0.05) in a multivariable model that adjusted for facility type, provider type, and years of experience offering EmONC services.

**Conclusions:**

Lack of equipment and training do not pose major barriers to newborn resuscitation in Afghanistan, but providers’ knowledge and skills need strengthening in some areas. Midwives proved to be as capable as doctors of performing newborn resuscitation, which validates the major investment made in midwifery education. Competency-based pre-service and in-service training, complemented by supportive supervision, is an effective way to build providers’ capacity to perform newborn resuscitation. This kind of training could also help skilled birth attendants based in the community, at private clinics, or at primary care facilities save the lives of newborns.

## Background

There were almost three million neonatal deaths worldwide in 2011; most were in low- and middle-income countries, which account for a growing proportion of all under-five mortality [1]. Some 23% of neonatal deaths can be attributed to intrapartum-related hypoxic events, formerly referred to as birth asphyxia [2]. Some of these deaths can be prevented by expanding and improving antenatal care, especially to detect and manage pre-eclampsia/eclampsia; skilled attendance at delivery; intrapartum care, including use of partographs; and emergency obstetrical care for complications [2]. However, approximately 3% to 6% of newborns require basic resuscitation, including stimulation at birth and assisted ventilation with bag and mask, to help them breathe [3]. This procedure can reduce intrapartum-related neonatal deaths by 30% [4]. There are no maternal risk factors in a substantial proportion of newborn resuscitation cases [5]. Hence, all skilled birth attendants (who are defined as doctors, midwives, and other health professionals trained to manage normal pregnancies and childbirth, to identify and manage complications in women and newborns, and to make needed referrals) should have the capacity to resuscitate newborn babies, whether deliveries take place in health facilities or at home.

Improving newborn health is a priority for the Ministry of Public Health (MoPH) in Afghanistan, where the neonatal mortality rate is currently estimated at 36 per 1000 live births [1]. The primary strategy for increasing access to health care in Afghanistan has been to implement a Basic Package of Health Services at lower-level facilities and an Essential Package of Hospital Services at district, provincial, regional, and specialized hospitals. The latter includes round-the-clock emergency obstetric and newborn care (EmONC). Newborn resuscitation is one of nine comprehensive EmONC services—called signal functions—that directly treat the obstetric and newborn complications that are the proximate causes of maternal and newborn death.^a^ To address high levels of mortality and morbidity among mothers and newborns in Afghanistan, efforts are being made to evaluate and scale up EmONC services, including intensive training to increase the number and capacity of providers [6].

The success of newborn resuscitation depends upon the knowledge and clinical skills of local birth attendants as well as access to basic equipment, including towels or blankets for drying, a bag and mask resuscitator, and a suction device. National surveys assessing the provision of health services in Africa and Asia have found that trained health workers and equipment for newborn resuscitation are not consistently available in all facilities [7,8]. Studies in diverse countries, including Cameroon, Ethiopia, Kenya, and Nepal, have identified missing equipment and inadequate provider knowledge and skills as barriers to the performance of newborn resuscitation [9-12].

Research has been limited, however, on providers’ actual performance of newborn resuscitation procedures and on individual or facility-level characteristics associated with that performance. In Ethiopia, a study found that recent performance of newborn resuscitation and geographic region were strong predictors of providers’ knowledge, but it did not assess how well providers’ knowledge translated into practical skills [9]. In Rwanda, an analysis of a national service provision assessment found no significant associations between provider or health system characteristics and the performance of recommended newborn care procedures, but it did not look at newborn resuscitation [13].

To investigate the capacity of EmONC facilities to perform newborn resuscitation and the factors that affect providers’ knowledge and skills, we conducted a further analysis of data collected by a national EmONC needs assessment in Afghanistan [14]. The objectives of the study are twofold: first, to determine whether facilities designated by the government to provide comprehensive EmONC services have the capacity (in terms of equipment and personnel) necessary to perform newborn resuscitation and, second, to identify which individual and facility-level characteristics are associated with providers’ knowledge of newborn resuscitation and clinical skills.

## Methods

A cross-sectional assessment of the availability and utilization of EmONC services in Afghanistan was conducted from December 2009 to February 2010 [15]. It examined all government health facilities designated to provide comprehensive EmONC services. These include district, provincial, regional, and specialized national hospitals. In remote areas that lack a referral hospital, certain Comprehensive Health Centers (CHCs) have been upgraded to provide comprehensive EmONC services; these facilities were also included in the needs assessment.

A total of 127 health facilities were expected to provide comprehensive EmONC services across Afghanistan’s 34 provinces. Of these, 49 facilities were inaccessible due to security constraints at the time of the assessment. Therefore, the assessment was limited to 78 facilities located in comparatively secure areas of the country. They included nine CHCs, 34 district hospitals, 25 provincial hospitals, five regional hospitals, and 5 specialized hospitals. Two-thirds of the facilities were located in urban areas. At provincial, regional, and specialized hospitals, two doctors and two midwives responsible for providing EmONC services were randomly selected to participate in the study. At district hospitals and CHCs, one doctor and one midwife were selected; if no doctor was available, a second midwife was chosen to participate. The total sample included 82 doctors and 142 midwives. Various kinds of doctors, including obstetrician/gynecologists, pediatricians, and general medical doctors, may perform newborn resuscitation at EmONC facilities [14].

The assessment team consisted of 6 doctors and 38 midwives. All were experienced service providers and had helped collect data for previous studies in Afghanistan. The assessors attended one week of training, after which intra- and inter-assessor reliability was tested. Assessors visited each facility for one to three days to collect data. Health facilities were not informed in advance about the assessors’ visit. Upon arrival at a facility, assessors obtained consent from the facility’s Medical Director and held an introductory meeting with key informants, including staff in charge of maternity, surgery, pharmacy, and laboratory departments.

To investigate the facility’s capacity to offer EmONC services, assessors conducted observations, interviewed key informants, and reviewed records for information on human resources, supplies, equipment, infrastructure, and services offered. They used modified tools based on the Averting Maternal Death and Disability Program’s Needs Assessment Toolkit [16]. To investigate providers’ proficiency in newborn resuscitation, the assessors employed two complementary methods. First, they interviewed all selected providers using a knowledge questionnaire based on model assessment forms in the Needs Assessment Toolkit. Assessors posed five open-ended questions regarding the steps providers should take during newborn resuscitation. Each question was designed to elicit multiple responses. The analysis looked at the proportion of providers who, without prompting, gave each of the 21 possible correct answers. Second, assessors observed all selected providers performing newborn resuscitation on an anatomical model and completed a 39-item skills checklist developed by Afghan midwifery schools to assess providers’ performance during clinical simulations.

All of the assessment tools were adapted for this study by the MoPH’s Reproductive Health Task Force and then reviewed, revised, and approved during a workshop involving the MoPH, national EmONC trainers, UNICEF, the World Health Organization, and experts from non-governmental organizations (NGOs) contracted by the MoPH to operate primary health facilities offering the Basic Package of Health Services. The tools were pilot tested during the assessors’ training workshop. The study protocols were approved by the institutional review boards of the Afghanistan Public Health Institute and the Johns Hopkins School of Public Health (IRB 2333 and IRB 2359).

Descriptive statistics were used to summarize facility and provider characteristics. A composite infrastructure score was created by calculating the percentage of 19 items present at each facility. Service records on the number of deliveries and neonatal deaths were compiled to estimate the early neonatal death rate. Student t-test or chi-square tests were performed to identify differences in knowledge and clinical skills between doctors and midwives. Pair-wise correlation and linear regression analyses were used to estimate the association of providers’ knowledge and clinical skills with facility characteristics (facility type, annual number of deliveries, number of newborn resuscitations performed, availability of guidelines, availability of supplies and equipment, and infrastructure) and provider characteristics (provider type, years of experience offering EmONC services, training on newborn resuscitation, number of newborn resuscitations performed in the past three months, and confidence in performing newborn resuscitation). A multivariable linear regression model was built to incorporate both facility and provider characteristics found significant in the bivariate analyses. Because key facility variables were closely correlated, only one was included in the final multivariable regression analysis: facility type. Three independent provider variables were used: provider type (doctor or midwife), training on newborn resuscitation, and years of experience offering EmONC services. Analyses were conducted using SAS (v.9.1.3, SAS Institute, Cary, NC) and STATA 11.2 (StataCorp, College Station, Texas) with a type 1 error of 0.05.

## Results and discussion

### Facility characteristics and caseload

Table 1 describes the 78 health facilities assessed. The government operated 90% of regional and specialized hospitals, but contracted out operation of 74.4% of CHCs and district hospitals to NGOs. Operation of provincial hospitals was evenly divided between the government and NGOs (48% and 52%, respectively). Caseloads were significantly higher at regional and specialized hospitals: on average, they reported more than four times as many deliveries annually as provincial hospitals and more than 11 times as many deliveries as district hospitals and CHCs (11,343; 2,546; and 946, respectively, p < 0.001). Caseloads also varied widely within facility types.

**Table 1:** **Characteristics of health facilities**, **by facility type**

Characteristics*	**CHCs and district hospitals (n = 43)**	**Provincial hospitals (n = 25)**	**Regional and specialized hospitals (n = 10)**	**p-value**
**Operating agency (%)**				
Government	25.6	48.0	90.0	**0.001**
Non-governmental organization	74.4	52.0	10.0	
**No. of deliveries in past 12 months**				
Range	38–3,775	164–11,771	1,989–23,924	
Mean (SD)	946 (905)	2,546 (2284)	11,343 (6,680)	**<0.001**
**At least one newborn resuscitation performed in past 3 months (%)**	86.0	100	100	0.071
**Guidelines available in the maternity ward (%)**				
Management of newborn complications	81.0	84.0	90.0	0.782
Postpartum/postnatal care of newborns	90.7	92.0	100.0	0.608
Immediate newborn care	88.4	92.0	100.0	0.500
**Availability of essential items (%)**				
Mucus extractor	95.2	96.0	100.0	0.783
Infant ambu bag	97.7	100.0	90.0	0.237
Infant face masks (sizes 0,1,2)	93.0	100.0	90.0	0.343
Towels or cloth for newborn	83.7	84.0	80.0	0.955
Newborn resuscitation table	72.1	83.3	90.0	0.703
**Availability of priority items (%)**				
Syringes (1 ml, 2 ml, 5 ml, 10 ml)	95.3	92.0	100.0	0.611
Suction apparatus	72.1	88.0	100.0	0.070
Stethoscope for use with newborns	53.4	52.0	90.0	0.085
Source of warmth	78.2	81.3	100.0	0.460
**Mean % (SD) of 19 components of infrastructure**^**a**^	66.6 (10.4)	74.2 (9.7)	79.5 (9.8)	**0.001**

*Items in bold reflect sub-categories of characteristics.

^a^Infrastructure components include electricity, generator, availability of water in different parts of facility, various kinds of telephones, radio, light sources, ventilation, toilet, heating, fan or air conditioning, curtains for patient privacy, and waiting area.

During the three months preceding the survey, all provincial, regional, and specialized maternity hospitals and 86% of CHCs and district hospitals had performed newborn resuscitation. The officer in charge of the maternity ward at six district hospitals and CHCs with low caseloads (from 276 to 1,126 deliveries annually) reported that newborn resuscitation had not been performed in the past three months, although registers and provider interviews suggest that they may have failed to recall some cases of newborn resuscitation (data not shown). One of these six facilities reported not performing the procedure due to lack of training, while the remaining five facilities reported that no newborns required resuscitation.

An analysis of service records showed that, across all 78 facilities, there were 7.4 early neonatal deaths (that is, newborn deaths occurring during the first 24 hours after delivery) per 1,000 births (Table 2). The early neonatal mortality rate was highest at provincial hospitals (12.7) and lowest at CHCs and district hospitals (2.9 and 1.3, respectively).

**Table 2:** **Very early neonatal mortality rate**, **by facility type**

**Facility type**	**Number of institutional deliveries**	**Number of newborn deaths occurring within 24 hours after delivery**	**Very early neonatal mortality rate (per 1000 deliveries)**
CHCs	2,770	8	2.9
District hospitals	36,068	47	1.3
Provincial hospitals	59,942	759	12.7
Regional hospitals	35,158	187	5.3
Specialized hospitals	58,689	421	7.2
*All facilities*	*192*,*627*	*1*,*422*	*7*.*4*

CHCs and district hospitals may experience lower neonatal mortality because they refer difficult cases to larger hospitals. They are also likely to receive women with less delay than provincial and regional hospitals. Even at referral hospitals, however, the death rate was not high. This may reflect the fact that two-thirds of women in Afghanistan deliver at home [6]; when complications occur, they may not get to a health facility in time, given long distances, difficult terrain, poor roads, and limited transportation [17]. However, the low early neonatal death rate also may reflect underreporting and misdiagnosis of early neonatal deaths as stillbirths. Distinguishing between a neonatal death and a stillbirth depends on providers’ knowledge and decision-making ability. There can be uncertainty surrounding the signs of life in a newborn and the need to resuscitate. Providers’ inability to recognize the need for resuscitation may help explain why five facilities reported no need for newborn resuscitation in recent months. Clinical audits could shed light on the causes of deaths occurring around delivery and indicate the extent of this problem in Afghanistan.

### Supplies and equipment for newborn resuscitation

Facilities were generally well-equipped, and there was no significant difference in the availability of any supplies or equipment by facility type (Table 1). Guidelines on newborn care were available at 81% to 100% of facilities. From 90% to 100% of facilities had a newborn-sized bag and mask and mucus extractor. Fewer facilities (from 72.1% to 90%) had a newborn resuscitation table. All regional and specialized hospitals had a suction apparatus, compared with 72.1% of CHCs and district hospitals and 88% of provincial hospitals. The proportion of basic infrastructure components in place at CHCs and district hospitals was significantly smaller than at provincial hospitals or regional and specialized hospitals (66.6%, 74.2%, and 79.5%, respectively, p < 0.001).

The importance of having newborn resuscitation equipment cannot be overemphasized: the United Nations Commission on Life-Saving Commodities for Women and Children includes a bag and mask device for newborn resuscitation on its list of 13 affordable, effective, but underutilized life-saving commodities [18]. In many resource-poor or low-income countries, especially in sub-Saharan Africa, the lack of these essential supplies poses a major barrier to performing effective newborn resuscitation [7]. In Afghanistan, however, nearly all of the facilities visited possessed these supplies, as well as most basic infrastructure components. However, a few facilities did lack a bag, mask, or simple suction device, and these deficiencies need to be addressed urgently. Other equipment is not as critical. Newborn resuscitation can be performed without a special resuscitation table, and simple newborn corners can be constructed with locally available materials to provide warmth and light for babies immediately after birth.

### Experience and training of providers

A total of 82 doctors and 142 midwives participated in the study. All were female, by cultural preference. Most (69.6% of doctors and 77.5% of midwives) worked at district or provincial hospitals (Table 3). Doctors and midwives did not differ significantly in the average number of years they had offered EmONC services (6.5 and 5.9 years, respectively), the proportion who had received training in newborn resuscitation (80.8% and 82.7%), or the proportion who felt very confident in their ability to perform the procedure (75.4% and 82.7%). However, doctors were significantly less likely than midwives to have received pre-service training in newborn resuscitation (35.4% and 59.2%, p < 0.001).

**Table 3:** **Characteristics of health care providers and their training**, **by provider type**

Characteristic*	**Doctors (n = 82)**	**Midwives (n = 142)**	**p-value**
**Location of work (%)**			
Comprehensive health center	7.3	7.8	0.479
District hospital	26.0	34.5	
Provincial hospital	43.9	43.0	
Regional hospital	10.9	7.8	
Specialized hospital	12.2	7.0	
**Years of experience offering emergency obstetric and newborn care: mean (SD)**	6.5 (5.5)	5.9 (6.8)	0.781
**Has received any training in newborn resuscitation (%)**	80.8	82.7	0.718
**Type of training received on newborn resuscitation (%)**^**a**^			
Pre-service	35.4	59.2	**0.001**
In-service	79.1	65.9	0.162
**Confidence in performing newborn resuscitation (%)**			
Very confident	75.4	82.7	0.232
Somewhat confident (needs coaching)	18.8	9.8	
Not confident	5.8	7.5	

*Items in bold reflect sub-categories of characteristics.

^a^Does not sum to 100% because providers may receive both pre-service and in-service training.

Findings on pre-service training reveal a fundamental difference in how midwives and doctors are trained. Newborn resuscitation is considered an essential midwifery competency [19] and has been part of the national midwifery curriculum in Afghanistan since 2004. Midwifery students must demonstrate their competency in performing resuscitation with actual newborns before they graduate, after first training with anatomical models, case studies, role plays, and/or clinical simulations. In contrast, pre-service education on newborn resuscitation offered to medical students is not competency-based. For the most part, students have to pass a written test on the subject; they receive little credit for demonstrating their skills on models or patients. Incorporating competency-based training on newborn resuscitation into the pre-service medical curriculum, as part of essential newborn care, should be a priority.

Once midwives and doctors enter service, they receive similar training on newborn resuscitation because the MoPH supports a team approach to in-service training on all EmONC signal functions. By helping different types of health professionals understand each other’s roles, team-based learning promotes greater collaboration and more effective health care [20]. In-service training on newborn resuscitation employs a competency-based approach with both midwives and doctors; it follows the same evidence-based standards for service delivery as pre-service training and emphasizes skill development. Job aids complement the training materials.

### Providers’ knowledge and clinical skills

Doctors and midwives displayed similar levels of knowledge on newborn resuscitation. On average, doctors gave 70.5% of correct answers and midwives 66% (Table 4). Doctors and midwives generally shared the same strengths and weaknesses. There was a significant difference between doctors and midwives on just 2 of 21 items: doctors were more likely than midwives to mention the need to position the baby’s head so the neck is slightly extended (89.5% and 69.9%, respectively, p < 0.001) and to administer oxygen if the baby does not begin breathing or experiences respiratory difficulty (92.3% and 79.7%, p < 0.05). Only a small proportion of doctors and midwives mentioned the need to explain what is happening to the mother either during resuscitation (23.4% and 24.1%) or after resuscitation (36.4% and 30.1%). Other common gaps in knowledge were: pausing to determine whether the baby is breathing spontaneously during resuscitation (44.2% of doctors and 40.9% of nurses mentioned this) and assessing the need for special care if the baby does not begin breathing or has respiratory difficulty after resuscitation (36.8% and 40.9%).

**Table 4:** **Provider knowledge**: **percent of providers who give correct responses to questions on newborn resuscitation**, **by provider type**

Questions and correct responses*	**Doctors (n = 82)**	**Midwives (n = 139)**	**p-value**
**How would you diagnose birth asphyxia?**			
Depressed breathing	83.3	76.9	0.263
Heart rate below 100 beats per minutes	56.6	50.8	0.416
Central cyanosis (blue tongue)	92.3	89.6	0.508
*Mean* (*SD*) (*3 items*)	*57* (*22*.*0*)	*54*.*8* (*19*.*3*)	*0*.*487*
**What are the preliminary steps of newborn resuscitation?**			
Place newborn face up	74.5	78.2	0.524
Wrap or cover baby	80.5	71.6	0.152
Position head so neck is slightly extended	89.5	69.9	**0.001**
Aspirate mouth and then nose	87.0	82.8	0.421
Explain to mother what is happening	23.4	24.1	0.911
*Mean* (*SD*) (*5 items*)	*69*.*5* (*23*.*0*)	*66*.*1* (*25*.*1*)	*0*.*307*
**What do you do when resuscitating with a bag and mask or tube and mask?**			
Place mask to cover chin, mouth and nose	88.5	91.8	0.424
Ensure seal between mask and face	72.7	64.7	0.229
Ventilate 1 or 2 times and see if chest is rising	66.2	57.9	0.233
Ventilate 40 times per minute for 1 minute	71.1	72.2	0.862
Pause to determine whether baby is breathing spontaneously	44.2	40.9	0.647
*Mean* (*SD*) (*5 items*)	*66*.*9* (*28*.*3*)	*65*.*7* (*29*.*5*)	*0*.*794*
**What do you do if the baby is breathing and there is no sign of respiratory difficulty?**			
Keep baby warm	87.2	81.1	0.250
Initiate breastfeeding	81.6	78.8	0.629
Continue monitoring the baby	66.7	62.4	0.534
*Mean* (*SD*) (*3 items*)	*74*.*0* (*30*.*6*)	*73*.*9* (*31*.*5*)	*0*.*993*
**What do you do if the baby does not begin breathing, breathing is less than 30 per minute, or if there is intercostal retraction or grunting?**			
Continue to ventilate	71.1	75.6	0.475
Administer oxygen, if available	92.3	79.7	**0.015**
Assess the need for special care	36.8	40.9	0.563
Explain to mother what is happening	36.4	30.1	0.348
*Mean* (*SD*) (*4 items*)	*57*.*6* (*24*.*4*)	*56*.*5* (*25*.*7*)	*0*.*752*
**Overall knowledge of newborn resuscitation (all 21 items)**	70.5	66.0	0.588

*Items in bold reflect sub-categories of questions, and italics the overall category or sub-category mean score.

Doctors and midwives demonstrated similar levels of clinical skill when simulating newborn resuscitation on an anatomical model; they completed 66.3% and 70.5%, respectively, of all tasks (Table 5). Doctors and midwives shared the same strengths and weaknesses, with midwives significantly outperforming doctors on just one skill: placing disposable suction catheters and mucus extractors in a leak-proof container (74% and 60%, p < 0.05). Providers of all kinds performed poorly on interpersonal communication: only 47.1% of doctors and 52.4% of midwives told the mother what was being done and responded to her questions and concerns. Even fewer offered emotional support and reassurance (41.7% and 46.0%). On average, doctors and midwives completed fewer post-procedure tasks (57.5% and 65.7%) than preparatory tasks (71.3% and 74.6%) or resuscitation tasks (71% and 74.6%).

**Table 5:** **Provider skills**: **percent of providers who complete specific tasks during simulation of newborn resuscitation on an anatomical model**, **by provider type**

Tasks*	**Doctors (n = 82)**	**Midwives (n = 139)**	**p-value**
**Getting ready**			
Make sure equipment is ready for use	91.7	91.3	0.937
Wash hands and wear gloves	69.0	79.5	0.098
Quickly dry and wrap or cover the newborn	91.7	92.1	0.909
Place newborn on back on clean, warm surface	86.1	87.4	0.795
Tell woman what is going to be done, listen to her, and respond to her questions and concerns	47.1	52.4	0.482
Provide emotional support and reassurance	41.7	46.0	0.552
*Mean* (*SD*) (*6 items*)	*71*.*3* (*27*.*1*)	*74*.*6* (*24*.*6*)	*0*.*379*
**Resuscitation using bag and mask**			
Position head in slightly extended position	91.7	88.3	0.453
Suction first the mouth and then the nose	90.3	89.1	0.788
Introduce catheter into mouth and suction	69.4	59.7	0.172
Introduce catheter into each nostril and suction	65.3	61.6	0.607
Suction well if blood or meconium is in the newborn’s mouth and/or nose	70.6	67.8	0.688
If baby is still not breathing, start ventilating	65.7	79.3	0.403
Recheck position of newborn’s head	68.1	72.4	0.513
Place correct-sized mask on newborn’s face	87.5	85.7	0.725
Form a seal between mask and newborn’s face	84.3	81.6	0.635
Squeeze bag	73.2	79.5	0.311
Check seal by ventilating and observing chest rise	83.1	77.8	0.373
If the newborn’s chest IS rising:			
Ventilate at 40 breaths/minute	87.5	81.0	0.234
Observe chest for easy rise and fall	83.3	75.4	0.193
If the newborn’s chest IS NOT rising:			
Check position of the head again	63.9	65.1	0.866
Reposition mask to improve seal	61.1	68.8	0.273
Squeeze the bag harder; repeat suction	56.9	58.4	0.842
Ventilate for 1 minute and then assess if the newborn is breathing spontaneously	71.8	75.8	0.541
If breathing is normal (no indrawing or grunting):			
Place in skin-to-skin contact with mother	87.5	81.0	0.079
Observe breathing at frequent intervals	83.3	75.4	0.868
Encourage mother to begin breastfeeding	63.9	65.1	0.174
If newborn is breathing with severe indrawing:			
Ventilate with oxygen, if available	61.1	68.8	0.893
Arrange immediate transfer for special care	56.9	58.4	0.492
If there is no gasping or breathing at all after 20 minutes of ventilation, stop ventilating	71.8	75.8	0.868
*Mean* (*SD*) (*23 items*)	*71*.*0* (*24*.*9*)	*74*.*6* (*24*.*0*)	*0*.*950*
**Post**-**procedure tasks**			
Place disposable suction catheters and mucus extractors in leak-proof container	60.0	74.0	**0.042**
For reusable catheters and mucus extractors:			
Place in chlorine solution for 10 minutes	65.7	76.8	0.095
Wash in water and detergent	66.2	70.6	0.518
Use a syringe to flush catheters/tubing	52.9	56.0	0.672
Boil or disinfect in chemical solution	40.8	53.6	0.086
Take apart valve/mask and inspect for cracks/tears	32.4	42.1	0.181
Wash valve/mask and check for damage	57.1	69.6	0.080
Select sterilization or high-level disinfection method	56.5	56.8	0.970
Wash hands and dry with clean cloth or air dry	77.5	85.2	0.172
After chemical disinfection, rinse all parts with clean water and allow to air dry	67.1	70.8	0.587
*Mean* (*SD*) (*10 items*)	*57*.*5* (*30*.*7*)	*65*.*7* (*29*.*3*)	*0*.*062*
**All tasks (39 items)**	66.3 (22.4)	70.5 (20.9)	0.185

*Items in bold reflect sub-categories of tasks, and italics the overall category or sub-category mean score.

Overall knowledge of newborn resuscitation was significantly lower among providers working at CHCs and district hospitals (57.9%) than providers working at provincial hospitals (67.8%) or regional and specialized hospitals (67.9%) (p < 0.01, data not shown). However, there was no significant difference in providers’ clinical skills at CHCs and district hospitals (65.9%), provincial hospitals (71.3%), and regional and specialized hospitals (70%).

The findings suggest that both doctors and midwives have the knowledge and skills needed to perform newborn resuscitation when indicated. Indeed, midwives proved to be equally knowledgeable, skilled, and confident as doctors in performing newborn resuscitation. Doctors and midwives also shared the same strengths and weaknesses on the knowledge and skills assessments, likely because they receive similar in-service training on newborn resuscitation. Given the strong correlation between competence and confidence in performing a skill [21], it is not surprising that the confidence of both doctors and midwives was generally high. The findings affirm the contribution that midwives are making to improve maternal and newborn health in Afghanistan. The country has made a significant investment in educating and graduating competent professional midwives, with over 3,000 midwives trained to date [22]. The MoPH has ensured that the duration and content of the new midwifery programs are extensive enough to permit the development of a professional cadre for women. The educational system, which includes a national mechanism to accredit midwifery schools and ensure quality education [23], also ensures that midwives in Afghanistan are uniquely positioned to provide life-saving care to newborns.

However, the findings reveal certain weaknesses in the knowledge and clinical skills of both midwives and doctors. These include interpersonal communication and post-procedure tasks related to infection prevention, even though both of these areas are integrated into training checklists and are considered to be essential in achieving competency in newborn resuscitation.

Supportive supervision and continuing professional education (still in its infancy in Afghanistan) could address these performance gaps, increase competence in weak skills, and foster providers’ confidence. However, an evaluation of midwifery education in Afghanistan found that both were lacking; practicing midwives and key informants called for a system of supportive supervision and regular refresher training to improve the quality of care [24]. This will require a fundamental change in the role of supervisors, who currently focus on documentation and paperwork and lack the expertise to serve as role models for providers. Supervisors should be encouraged to observe providers with patients, offer constructive feedback and instruction on good care, and enlist management support.

Maintaining competence is especially challenging in facilities with low caseloads and therefore limited opportunities to practice newborn resuscitation. This can be overcome by a combination of periodic refresher training and regular practice with anatomical models, which can easily be transported to clinical sites [7]. Onsite refresher training is more sustainable than external workshops and also prevents staff shortages that result when providers must travel long distances to attend training courses [25]. Afghanistan is one of many countries embarking on the Helping Babies Breathe program (http://www.helpingbabiesbreathe.org), which offers simplified but frequent training designed to improve provider performance in newborn resuscitation.

Supervision and in-service training may not be sufficient to address all of the performance gaps identified, especially weaknesses in referrals and interpersonal communication skills. Many providers did not recognize the need to arrange for transfers when newborns need special care after resuscitation. This likely reflects broader problems facing the health system in Afghanistan; geographical, security and climatic challenges exacerbate gaps in coverage and constraints on ensuring an effective referral system. One particular problem is that lower-level facilities do not receive any information regarding outcomes for patients referred to higher-level facilities, even whether they live or die. Without this kind of feedback, there can be no accountability. As for interpersonal communication skills, providers simply do not understand that communication is a vital component of woman-centered, respectful care [26]. A fundamental change is required in providers’ attitudes towards patients, which must begin during pre-service education. Once the foundation is laid in pre-service education, in-service training and supportive supervision can then reinforce efforts to ensure that women and their families are fully informed about a newborn’s condition.

### Factors associated with knowledge and skills

Bivariate analyses examined which facility and provider characteristics were associated with providers’ knowledge and clinical skills. Providers’ knowledge of newborn resuscitation was significantly associated with facility type, infrastructure, and three provider characteristics: years of experience offering EmONC services, training on newborn resuscitation, and confidence in performing newborn resuscitation (Table 6). Clinical skills were associated with just two provider characteristics: training on newborn resuscitation and confidence in performing newborn resuscitation.

**Table 6:** **Univariate analysis**: **association between provider and facility characteristics and provider knowledge and clinical skills in newborn resuscitation**

Characteristic*	**Overall knowledge**			**Overall clinical skills**		
	**Beta**	**SE**	***P***	**Beta**	**SE**	***P***
**Facility characteristics**						
Facility type^1^						
Provincial hospital	0.50	0.19	**0.008**	0.19	0.14	0.18
Regional or specialty hospital	0.55	0.24	**0.024**	0.15	0.18	0.41
Annual number of deliveries:^2^						
Second quintile	0.01	0.27	0.96	0.03	0.19	0.86
Third quintile	0.15	0.27	0.57	−0.07	0.19	0.71
Fourth quintile	0.23	0.28	0.40	0.20	0.19	0.23
Highest quintile	0.23	0.39	0.48	0.08	0.20	0.68
Newborn resuscitation performed in past three months	−0.09	0.52	0.87	0.12	0.26	0.64
Availability of guidelines	0.09	0.11	0.44	−0.06	0.08	0.50
Availability of supplies and equipment	0.05	0.03	0.10	0.02	0.02	0.31
% of infrastructure components	0.11	0.04	**0.006**	−0.004	0.03	0.89
**Provider characteristics**						
Type of provider	0.04	0.12	0.74	−0.12	0.08	0.13
Years of experience offering EmONC	−0.03	0.01	**0.006**	−0.003	0.007	0.69
Number of newborn resuscitations performed in past three months	0.001	0.001	0.55	0.002	0.001	0.06
Newborn resuscitation training received	0.64	0.17	**<0.001**	0.36	0.11	**0.002**
Confidence in performing resuscitation^3^						
Very confident	1.81	0.25	**<0.001**	0.84	0.23	**<0.001**
Somewhat confident, needs coaching	1.48	0.25	**<0.001**	0.74	0.27	**0.007**

^1^Reference group is CHCs and district hospitals.

^2^Quintile 1 (lowest) is the reference quintile. Quintile cut-offs are: 547, 1268, 1617, and 4283.

^3^Reference group is “no confidence”.

The multivariable linear regression analysis controlled for facility type, type of provider, training in newborn resuscitation, and years of experience offering EmONC services. The results show that working at provincial hospitals is positively associated with providers’ knowledge (p < 0.05), but not their clinical skills. Training is associated with both knowledge (p < 0.001) and skills (p < 0.05), while provider type and clinical experience are associated with neither (Table 7).

**Table 7:** **Multivariable regression analysis of providers**’ **knowledge and skills**^**1**^

**Factors**	**Total knowledge score**			**Total skills score**		
	**Coeff.**	**SE**	***P***	**Coeff.**	**SE**	***P***
Facility type^2^						
Provincial hospital	0.45	0.19	**0.02**	0.17	0.13	0.20
Regional or specialty hospital	0.45	0.26	**0.08**	0.10	0.18	0.56
Type of provider	0.04	0.17	0.77	−0.12	0.08	0.14
Training in newborn resuscitation	0.58	0.17	**0.001**	0.27	0.12	**0.02**
Years of experience offering EmONC	−0.01	0.01	0.33	−0.004	0.006	0.48

^1^Final model included 210 providers for knowledge score and 188 for skill score.

^2^Reference group is CHCs and district hospitals.

The results of this analysis suggest that, among the variables examined, training exerts the greatest influence on providers’ knowledge and skills regarding newborn resuscitation. Other studies have drawn similar conclusions [10,12,27]. The analysis did not distinguish between pre-service and in-service training. However, doctors’ proficiency in newborn resuscitation, despite the limitations of their pre-service education, suggests that in-service training may be especially effective.

In contrast to training, providers’ experience (as measured by the number of years that they had offered EmONC services) was not associated with knowledge or clinical skills in the multivariable analysis. Experience may be more strongly related with confidence than either knowledge or skills. In actuality, the longer providers have been in practice, the more likely it is that standards of care have changed in the interim, leaving them out of date. Experienced providers may become so confident in their skill set that they are less adaptable and more reluctant to change.

Interestingly, the facility setting seems to have an impact on providers’ knowledge, but not skills. This likely reflects differences in the learning environment at EmONC facilities. Larger hospitals have specialist physicians and medical residents on staff and actively promote continuing education with grand rounds, talks, and meetings. In contrast, doctors and midwives who work at CHCs and district hospitals may have few, if any, colleagues and limited opportunities for learning; this makes in-service training activities especially important at smaller facilities.

### Methodological strengths and limitations

A central strength of this study is that it uses a variety of data collection methods to measure multiple components of the capacity for newborn resuscitation, including training, confidence in performing the procedure, having essential supplies on hand, and performing skills correctly. Thus, it can address two research gaps, the first on effective strategies for translating knowledge into practice in low-income countries [28] and the second on facility characteristics or other situational factors that may influence whether knowledge gained during training is maintained and applied. Understanding the potential impact of the facility setting and provider characteristics on newborn resuscitation can help inform continuing education and reinforcement activities and guide efforts to standardize resources at each level of facility across Afghanistan.

Interpretation of the findings is subject to certain limitations. First, although this study was designed to be national in scope, the number and location of facilities assessed was limited by the ongoing conflict and associated security concerns in parts of Afghanistan. Hence, the assessment does not provide a representative snapshot of conditions across the entire country. Second, the sampling strategy—which selected similar numbers of providers from each facility—underrepresents providers who work at larger, busier facilities and may have stronger skills. Third, a clinical simulation with anatomical models may not reflect providers’ actual performance on the job, including how quickly they act when newborn resuscitation is indicated. Fourth, contradictions between different information sources, e.g., interviews and registers, suggest that data on services offered at each facility may be subject to recall and reporting bias.

## Conclusions

Improving the quality of EmONC services is just one way to reduce neonatal deaths from intrapartum hypoxia in Afghanistan. Other interventions are also required. For newborns who have difficulty breathing, however, the key is ensuring that skilled birth attendants are present at every delivery and prepared to perform newborn resuscitation, no matter where the birth takes place—at home or in a private clinic, primary care facility, or EmONC facility. These providers do not necessarily need to be doctors. In this study, midwives proved as capable as doctors of performing newborn resuscitation, which validates the investment made in midwifery education in Afghanistan. Other studies have shown that training and equipping community health workers and traditional birth attendants to perform newborn resuscitation during home deliveries can save lives [4].

The findings suggest that expanding and strengthening competency-based pre-service and in-service training is the most effective strategy for building the capacity of skilled birth attendants to perform newborn resuscitation, as long as essential supplies and equipment are available. This supports recommendations on training and supplies made by Afghanistan’s Reproductive Health Department in its strategic planning document for 2012 – 2016 [6]. However, training alone will not be sufficient for providers who staff lower-level facilities or attend home births, because they do not see many cases requiring newborn resuscitation and have fewer opportunities to practice. Regular reinforcement of providers’ knowledge and skills should be a priority in these settings.

Tracking the proportion of providers who are proficient in newborn resuscitation and the proportion of newborns receiving resuscitation—and validating those data through facility assessments or retrospective surveys—is considered a priority for improving newborn survival [29-31]. This study demonstrates that simulations with an anatomical model are a practical way to assess newborn resuscitation skills and identify providers’ weaknesses. This approach offers a less costly and more practical alternative to direct observation of actual newborn resuscitation procedures, which requires observers to wait for newborn resuscitation cases to occur. The study also contributes to the development of a composite indicator to measure a facility’s capacity to conduct newborn resuscitation, one which includes supplies and equipment as well as provider proficiency.

### Endnotes

^a^There are seven basic EmONC signal functions: parenteral antibiotics, parenteral anticonvulsants, parenteral oxytocics, manual removal of placenta, removal of retained products, assisted or instrumental vaginal delivery, and newborn resuscitation. Comprehensive EmONC also includes blood transfusion and cesarean section.

## Competing interests

The authors declare that they have no competing interests.

## Authors’ contributions

YMK designed the study, served as the Primary Investigator, and coordinated the manuscript drafting and finalization process. NA participated in the design and implementation of the study, contributed to the analysis and interpretation of study findings, and revision of the manuscript. AK contributed to the interpretation of study findings and writing and revision of the manuscript. RS and KS conducted initial data analysis and contributed to writing the first draft of the manuscript. HT conducted subsequent data analysis and contributed to the interpretation of study findings and writing and revision of the manuscript. SC contributed to interpretation of study findings and writing of the discussion section. PB, JvR, and JS participated in critical review of the manuscript and provided key inputs into the discussion and conclusions. All authors read and approved the final manuscript.

## Pre-publication history

The pre-publication history for this paper can be accessed here:

http://www.biomedcentral.com/1471-2431/13/140/prepub
